# The "lipid accumulation product" performs better than the body mass index for recognizing cardiovascular risk: a population-based comparison

**DOI:** 10.1186/1471-2261-5-26

**Published:** 2005-09-08

**Authors:** Henry S Kahn

**Affiliations:** 1 National Center for Chronic Disease Prevention and Health Promotion, CDC, Mail-stop K-10, 4770 Buford Highway, Atlanta, Georgia 30341-3717 USA

## Abstract

**Background:**

Body mass index (BMI, kg/m^2^) may not be the best marker for estimating the risk of obesity-related disease. Consistent with physiologic observations, an alternative index uses waist circumference (WC) and fasting triglycerides (TG) concentration to describe lipid overaccumulation.

**Methods:**

The WC (estimated population minimum 65 cm for men and 58 cm for women) and TG concentration from the third National Health and Nutrition Examination Survey (N = 9,180, statistically weighted to represent 100.05 million US adults) were used to compute a "lipid accumulation product" [LAP = (WC-65) × TG for men and (WC-58) × TG for women] and to describe the population distribution of LAP. LAP and BMI were compared as categorical variables and as log-transformed continuous variables for their ability to identify adverse levels of 11 cardiovascular risk factors.

**Results:**

Nearly half of the represented population was discordant for their quartile assignments to LAP and BMI. When 23.54 million with ordinal LAP quartile > BMI quartile were compared with 25.36 million with ordinal BMI quartile > LAP quartile (regression models adjusted for race-ethnicity and sex) the former had more adverse risk levels than the latter (p < 0.002) for seven lipid variables, uric acid concentration, heart rate, systolic and diastolic blood pressure. Further adjustment for age did not materially alter these comparisons except for blood pressures (p > 0.1). As continuous variables, LAP provided a consistently more adverse beta coefficient (slope) than BMI for nine cardiovascular risk variables (p < 0.01), but not for blood pressures (p > 0.2).

**Conclusion:**

LAP (describing lipid overaccumulation) performed better than BMI (describing weight overaccumulation) for identifying US adults at cardiovascular risk. Compared to BMI, LAP might better predict the incidence of cardiovascular disease, but this hypothesis needs prospective testing.

## Background

Obesity is commonly understood to imply excess fat, but it is ordinarily classified according to excess weight. This semantic inconsistency may help to explain why the body mass index (BMI, kg/m^2^) – a popular marker for relative weight – performs only modestly as a predictor of medical risk [1]. Researchers have increasingly appreciated that adipose tissue has complex functions [2,3], and that these functions may vary according to anatomic region [4-8]. Some of the region-specific functions of adipose tissue are beneficial, such as storing and buffering daily fluxes of circulating lipid fuels [9]. Thus, one cannot assume that a high relative weight or global adiposity is always deleterious.

In the current era of increasing obesity, we should attempt to define and measure lipid accumulation specifically in those contexts where accumulation may represent a physiologic danger [10,11]. These contexts might be described as lipid ***over***accumulation [12]. At the same time we should avoid attributing culpability to components of adipose or lean tissue that are enlarged but might enhance physiologic processes or reduce the risk of disease.

This paper first describes a simple index for estimating lipid overaccumulation among adults. Next, to demonstrate the utility of the lipid overaccumulation concept, this paper tests the hypothesis that the described index is better correlated than BMI with a variety of cardiovascular risk factors. This hypothesis should not be surprising since the BMI can neither distinguish between fat and lean tissues nor identify the anatomic location or function of distinct fat depots.

The proposed index – designated the "lipid accumulation product" (LAP) – is based on a combination of two measurements that are safe and inexpensive to obtain. One is waist circumference (WC), a measure of truncal fat that includes the visceral (intra-abdominal) depot. The other is the fasting concentration of circulating triglycerides (TG), the esterified, long-chain fatty acids that circulate through blood contained stably inside lipoproteins. Both waist size and TG concentration tend to rise with age [13,14], suggesting that their values are subject to accumulation over time. Waist size and circulating TGs are each continuously associated with metabolic insulin resistance [15,16], daylong triglyceridemia [17], and cardiovascular risk [18-20].

Although previous papers have proposed that the combination of enlarged waist and elevated TGs might serve as a dichotomous risk marker [21,22], the simple index described here was developed to express a continuous risk function. If LAP is better correlated than BMI with cardiovascular risk factors, this finding would support the notion that the overaccumulation of lipid carries worse cardiovascular consequences than the less specific overaccumulation of weight.

## Methods

### Defining the lipid accumulation product (LAP)

Data were obtained from the third National Health and Nutrition Examination Survey (NHANES III), a probability sample of the US civilian, noninstitutionalized population that included an oversample of non-Hispanic blacks and Mexican Americans [23]. NHANES III, conducted over the period 1988–1994, was unique among large US surveys because it incorporated assays of serum apolipoproteins B and A1 (ApoB, ApoA1; restricted to survey years 1988–1991). The analytic population from the seven-year survey contained 4,447 male and 4,733 female participants who were aged 18+ years, not pregnant, had fasted 8–19 hours before their laboratory examination, and had data available on basic anthropometry and fasting serum TGs (excluding three persons with a TG concentration >15 mmol/L). Participants were asked to complete a household interview and a standardized examination, including measurement of the standing waist circumference (in the horizontal plane at the level just above the right iliac crest, at minimal respiration) [24,25]. Serum TGs were measured enzymatically after hydrolysis to glycerol (Hitachi 704 Analyzer; Boehringer Mannheim, Indianapolis, Indiana); the coefficient of variation was 3–5 percent over the study and across the clinical range. Additional details of all laboratory procedures are available elsewhere [26]. Calculations of low-density lipoprotein (LDL) cholesterol concentration were limited to participants with TG concentrations below 4.5 mmol/L (a requirement of the Friedewald equation [27]) who had fasted at least nine hours.

Sampling weights from NHANES III were used with the software programs SAS, SAS/Graph (Release 8.2, SAS Institute, Cary, NC), and SUDAAN (Release 8.0, Research Triangle Institute, Research Triangle, NC), to estimate the sizes of the represented adult populations, to describe the distributions in the population of risk factors associated with LAP and BMI, and to perform analyses using multivariable linear regression. The analyses thus incorporated sampling weights that accounted for unequal selection probabilities (clustered design, planned oversampling, and differential nonresponse) [28]. Based on the sampling weights assigned, the analytic cohort represented an estimated total of 100,048,439 US adults aged 18+ years, 50.5 percent (SE 0.7) of them women, with a distribution of race-ethnicity that was 76.0 (1.5) percent non-Hispanic white, 10.5 (0.6) percent non-Hispanic black, 5.3 (0.5) percent Mexican American, and 8.1 (1.1) percent other.

**Figure 1:**
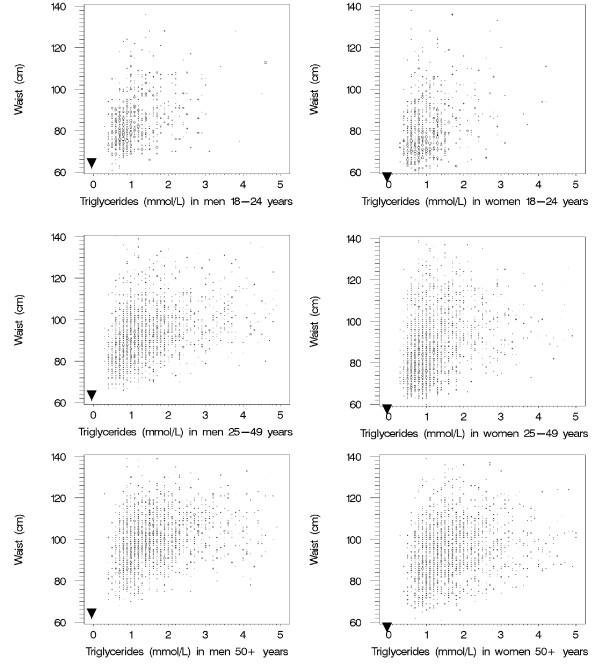
**Age-specific distributions by waist circumference and fasting triglyceride concentrations, from NHANES III. **For consecutive age groups the population density is increasingly displaced away from the hypothetical origin points of zero lipid accumulation (solid triangles). For clarity of presentation, the bubble plots omit the extreme outlying values for WC and TG.

Sex-specific bubble plots of population density by the values for WC (to the nearest cm) and TG concentration (to the nearest 0.1 mmol/L) were prepared to represent US adults in three age ranges (figure 1). The area of each bubble on these plots is proportional to the estimated number of men or women represented by those intersections. A sex-specific hypothetical minimum value for WC (that is, the waist size that theoretically contained only abdominal muscle, viscera, and vertebral bone) was estimated by calculating the mean minus two standard deviations of the log-transformed WC value among the estimated 15.00 million persons aged 18–24 years. These estimated minimum WC values (65 cm for men and 58 cm for women) were very similar to minimum values reported in a survey of 18 year-old Canadians in 1981 [29].

The minimum WC values were used to define sex-specific origin points (near the left-lower corner of each panel in figure 1) that represented a hypothetical state in which TG concentrations were arbitrarily set to zero and the waist size (greater for men than women) comprised primarily lean truncal tissue. A comparison of sex-specific bubble plots by age group confirmed that increasing age was accompanied by a shift of the population density upward (increasing waist size) and to the right (increasing circulating TG). Consistent with this empirical observation, the LAP was defined to describe the extent to which an individual had travelled the route – in theory – of both increasing waist and increasing TG:

LAP for men = (WC [cm] - 65) × (TG concentration [mmol/L])

LAP for women = (WC [cm] - 58) × (TG concentration [mmol/L])

In order to avoid having nonpositive values for LAP, any waist values for men that were 65 cm or less (five men in the NHANES III sample, all aged 18–22 years) were revised upward to 66.0 cm. No women in the entire NHANES III sample had a waist circumference less than 58.4 cm.

### Comparing LAP with BMI

#### Assessment of discordant subpopulations

Starting with the estimated full population, two subpopulations were identified for which the quartile classifications for LAP and BMI were discordant (table 1). The subpopulation whose ordinal LAP quartile was greater than their ordinal BMI quartile (i.e., those located below the bolded diagonal cells in table 1) was compared with the subpopulation whose ordinal LAP quartile was less than their ordinal BMI quartile (i.e., those located above the bolded diagonal cells). An estimated 48.91 million individuals (47.7 percent of the men, 50.1 percent of the women) were located in discordant quartiles. To calculate the effect of these discordant classifications on 11 cardiovascular risk variables, the two discordant subpopulations were compared in linear regression models using adjustments for sex and race-ethnicity and then again with further adjustment for age (terms for age and age^2^). All comparisons are reported with two-sided p values.

**Table 1:** Distribution of US adults by population quartiles of lipid accumulation product and body mass index. Table shows number of survey participants and corresponding population estimates (millions, in parentheses). Participants identified in the bold-print cells are concordant for their quartile assignment both to LAP and BMI.

	Quartiles of lipid accumulation product (LAP)					
		4	3	2	1	Total
Quartiles of body mass index (BMI)	4	**1543 (15.45)**	809 (7.30)	248 (2.26)	21 (0.17)	2621 (25.19)
	3	686 (6.93)	**983 (9.66)**	724 (7.28)	164 (1.42)	2557 (25.30)
	2	191 (2.23)	531 (6.08)	**742 (9.53)**	521 (6.93)	1985 (24.77)
	1	34 (0.39)	176 (1.95)	494 (5.97)	**1313 (16.49)**	2017 (24.79)
	Total	2454 (25.00)	2499 (24.99)	2208 (25.03)	2019 (25.02)	9180 (100.05)

#### Comparison of continuous linear regression models in the full population

Linear regression models were prepared from the entire population (discordant and concordant) that used either log-transformed LAP (ln LAP) or log-transformed BMI (ln BMI) as continuous independent variables. The dependent (outcome) variables were the same set of 11 cardiovascular risk factors. Models were also prepared separately for two age groups, those under age 50 years and those aged 50+ years, with adjustments for race-ethnicity and for sex when the sexes were combined. For each outcome risk variable, ln LAP and ln BMI were evaluated by comparing the proportion of the total variation that each index could explain, that is, R^2 ^for the entire model minus R^2 ^for a base model that excluded ln LAP and ln BMI. For these continuous analyses, the beta coefficients (slopes) were standardized to reflect the increment in each outcome variable associated with an increment of one standard deviation from the mean (calculated for each sex and age group [18–49 years or 50+ years]) of either ln LAP or ln BMI.

## Results

### Distributions of LAP and BMI

The population-based distributions of LAP and BMI were skewed to the right (table 2), but with logarithmic transformation both indices approached a normal distribution within each age group. Mean and median values rose consistently with age for both LAP and BMI. However, for LAP (more than for BMI) the values for men rose more rapidly before age 50 years. Above that age the sex differences were attenuated. Within the middle age range (25–49 years) the upper quartile of men's LAP was greater than the women's upper quartile, but for the upper quartile of BMI the sex contrast went in the opposite direction.

**Table 2:** Population estimates of lipid accumulation product and body mass index by sex and age group. Estimates derived for US adults from NHANES III, 1988–1994.

			Lipid accumulation product (LAP) *cm·mmol/L*				Body mass index (BMI) *kg/m*^2^		
			P e r c e n t i l e				P e r c e n t i l e		
Sex & age	Survey sample (N)	Geometric mean	25^th^	50^th^	75^th^	Geometric mean	25^th^	50^th^	75th
Men									
18–24 years	685	16.2	9.2	15.5	27.6	23.6	21.1	23.1	25.7
25–49 years	1982	35.0	20.1	35.5	63.2	26.4	23.7	25.9	29.0
50+ years	1780	52.4	33.3	53.4	85.6	26.9	24.3	26.8	29.9
Women									
18–24 years	715	16.6	9.4	16.0	27.6	23.1	20.0	22.4	25.6
25–49 years	2216	25.7	13.6	24.6	47.7	25.4	21.5	24.6	29.5
50+ years	1802	50.2	29.6	51.7	84.5	26.9	23.1	26.4	30.7
All									
18–24 years	1400	16.4	9.2	15.7	27.6	23.4	20.6	22.8	25.6
25–49 years	4198	30.1	16.4	29.7	57.3	25.9	22.5	25.3	29.1
50+ years	3582	51.2	31.1	52.6	85.3	26.9	23.8	26.6	30.3

**Figure 2:**
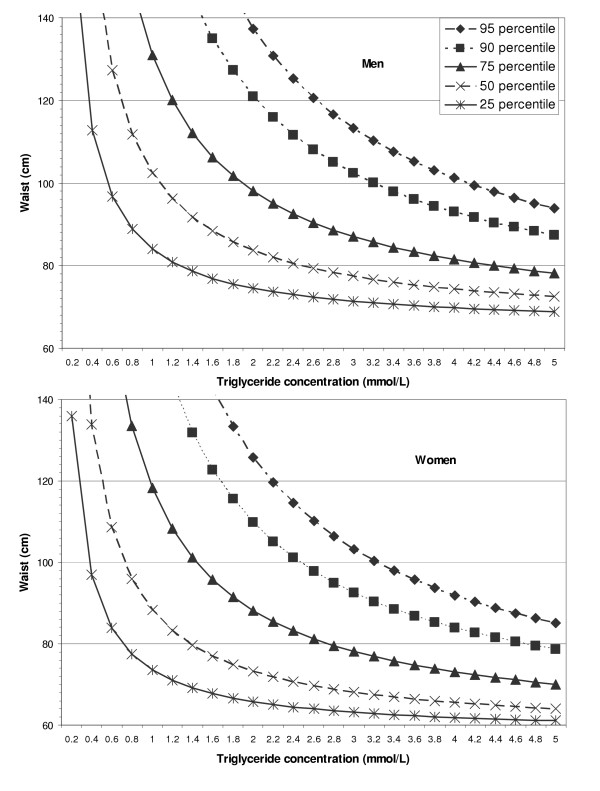
**Lines of equivalent percentile value for the lipid accumulation product (LAP) among US adults. **Population estimates from NHANES III (USA, 1988–1994) are shown separately for men (top panel) and women (bottom panel). The presented iso-LAP values (95th through 25th percentiles) are 144.7, 112.0, 66.1, 37.4, and 19.1 cm·mmol/L for men and 135.6, 103.5, 60.4, 30.3, and 15.6 cm·mmol/L for women.

Applied to the entire adult age range, the male and female quartile cutpoints for LAP were similar, although the men's values were slightly higher than the women's (figure 2). For BMI, the quartile cutpoints were higher for men than women at the 25th and 50th percentiles (23.3 and 25.7 vs. 21.7 and 24.8 kg/m2), but at the 75th percentile the BMI cutpoint was lower for men than women (28.9 vs. 29.6 kg/m2).

The linear correlation between LAP and BMI for the adult population was modest (r = 0.58) and somewhat stronger when both indices were log-transformed (r = 0.71).

### Comparisons of LAP with BMI regarding cardiovascular risk variables

#### Analyses restricted to discordant subpopulations

Compared to the subpopulation with ordinal BMI quartile > ordinal LAP quartile, the subpopulation with ordinal LAP quartile > ordinal BMI quartile was older [50.5 (SE 0.7) years vs. 38.5 (0.6) years], had more non-Hispanic whites [82.2 (1.8) vs. 69.4 (2.0) percent], and fewer non-Hispanic blacks [5.2 (0.5) vs. 17.5 (1.3) percent] and Mexican Americans [4.4 (0.5) vs. 5.9 (0.6) percent]. In regression models adjusted for sex and race-ethnicity (table 3), they had more adverse levels for all 11 of the evaluated cardiovascular risk factors (p < 0.002). These included the two factors for which mean values are inversely associated with risk – concentration of high-density-lipoprotein (HDL) cholesterol and ratio of low-density-lipoprotein (LDL) cholesterol to apolipoprotein B (i.e., smaller size of LDL particles [30]). With additional adjustment for age, the relatively adverse status of higher LAP was no longer seen for systolic and diastolic blood pressure (p > 0.1), but it remained (p < 0.0005) for all seven lipid variables, uric acid concentration, and heart rate (table 3, right columns). The exclusion of persons who reported that they were taking medicine prescribed for high blood pressure (prevalence 11.3 percent) or to lower their cholesterol (prevalence 2.8 percent) did not alter any of these observed relationships (data not shown).

**Table 3:** Mean levels of cardiovascular risk variables among the subpopulations discordant for quartiles of LAP and BMI. Estimates derived for US adults from NHANES III, 1988–1994.

		Mean (SE) adjusted for sex and race-ethnicity			Mean (SE) adjusted for sex, race-ethnicity, and age		
					
Dependent variable, *units*	Discordant survey sample (N)	LAP quartile > BMI quartile	BMI quartile > LAP quartile	P value	LAP quartile > BMI quartile	BMI quartile > LAP quartile	P value
Total cholesterol, *mmol/L*	4598	5.62 (0.04)	4.89 (0.04)	<0.0001	5.50 (0.04)	5.00 (0.04)	<0.0001
HDL cholesterol, *mmol/L*	4578	1.24 (0.01)	1.37 (0.01)	<0.0001	1.22 (0.01)	1.39 (0.01)	<0.0001
LDL cholesterol, *mmol/L*	3333	3.50 (0.04)	3.11 (0.05)	<0.0001	3.40 (0.04)	3.20 (0.05)	0.0003
Total cholesterol / HDL cholest.	4577	4.93 (0.06)	3.78 (0.05)	<0.0001	4.88 (0.06)	3.82 (0.05)	<0.0001
Apolipoprotein B, *g/L*	2245*	1.13 (0.01)	0.94 (0.01)	<0.0001	1.10 (0.01)	0.97 (0.01)	<0.0001
ApoB/ApoA1	2230*	0.809 (0.012)	0.681 (0.010)	<0.0001	0.799 (0.013)	0.690 (0.010)	<0.0001
LDL cholesterol /ApoB, *mmol/g*	1614*	3.06 (0.04)	3.26 (0.03)	0.0007	3.03 (0.04)	3.29 (0.03)	<0.0001
Uric acid, *mmol/L*	4533	327 (2)	311 (3)	<0.0001	325 (2)	313 (3)	0.0004
Systolic blood pressure, *mmHg*	4595	124.9 (0.7)	118.3 (0.4)	<0.0001	121.3 (0.6)	121.6 (0.4)	0.62
Diastolic blood pressure, *mmHg*	4595	74.5 (0.3)	73.0 (0.3)	0.0018	74.1 (0.3)	73.4 (0.3)	0.14
Heart rate, *bpm*	4494	75.0 (0.5)	72.4 (0.6)	0.0005	75.1 (0.5)	72.3 (0.6)	0.0001

* Lipoprotein data obtained only during phase 1 of NHANES III.

#### Analyses of continuous linear regression models in the full population

**Figure 3:**
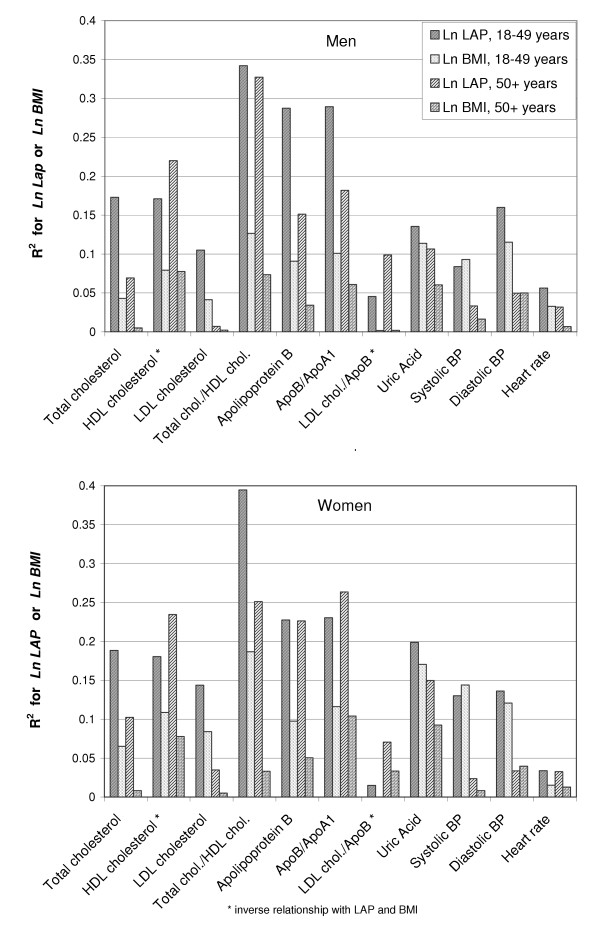
**Proportion of total population variation (R^2^) in risk variables explained by *Ln LAP *and *Ln BMI*. **Histograms were estimated from NHANES III data (USA,1988–1994) showing R^2 ^values for sex-specific, age-specific, regression models of cardiovascular risk variables after adjustment for race-ethnicity.

Ln LAP consistently explained a greater portion of the variation of the outcome variables than did ln BMI for all seven lipid outcome variables, uric acid concentration, and heart rate (figure 3). For most of these variables the proportion of total variance explained (R^2^) by ln LAP was about twice that of ln BMI. For the remaining two variables, systolic and diastolic blood pressure, the contrasts in portion of explained variation were small but consistent between the sexes. In the population under age 50 years, ln BMI was the better predictor of systolic blood pressure, but ln LAP was better for diastolic pressure. In the population aged 50+ years, ln LAP was the stronger predictor of systolic blood pressure.

A similar pattern was seen in a comparison of the standardized beta coefficients applied to the entire adult age range (data not shown). The beta coefficient (slope) of ln LAP was consistently more adverse (p < 0.01) than that of ln BMI for nine of the cardiovascular risk variables, but not for systolic and diastolic blood pressures (p > 0.2). When these comparative models were stratified by the two age groups the relatively greater slope of ln LAP was preserved among the nine variables for both age groups (table 4). Again, the exclusion of self-reported medication users did not alter these relationships (data not shown).

**Table 4:** Estimated increments in each risk variable per 1 standard deviation of ln LAP or ln BMI. Population estimates derived for US adults from NHANES III (1988–1994) with adjustments for sex and race-ethnicity.

Dependent variable	Age (years) range	Mean outcome value, *units*	Observations (N) used in analyses	Increment (SE) per 1 SD in ln LAP	Increment (SE) per 1 SD in ln BMI
Total cholesterol	18–49	4.96 *mmol/L*	5597	0.413 (0.023)	0.225 (0.018)***
	50+	5.75 *mmol/L*	3582	0.320 (0.024)	0.089 (0.022)***
HDL cholesterol	18–49	1.29 *mmol/L*	5571	-0.152 (0.008)	-0.111 (0.007)**
	50+	1.32 *mmol/L*	3570	-0.187 (0.009)	-0.109 (0.010)***
LDL cholesterol	18–49	3.09 *mmol/L*	3858	0.326 (0.023)	0.220 (0.021)**
	50+	3.62 *mmol/L*	2754	0.146 (0.031)	0.058 (0.026)
Total cholesterol/HDL cholest.	18–49	4.16 *mmol/L*	5570	0.905 (0.040)	0.584 (0.033)***
	50+	4.74 *mmol/L*	3570	0.930 (0.042)	0.383 (0.042)***
Apolipo-protein B	18–49	0.97 *g/L*	2668†	0.123 (0.006)	0.074 (0.007)***
	50+	1.16 *g/L*	1807†	0.115 (0.010)	0.056 (0.007)***
ApoB/ApoA1	18–49	0.717	2642†	0.115 (0.006)	0.074 (0.006)***
	50+	0.810	1801†	0.109 (0.009)	0.069 (0.006)**
LDL cholesterol/ApoB	18–49	3.15 *mmol/g*	1813†	-0.090 (0.017)	-0.013 (0.012)**
	50+	3.19 *mmol/g*	1393†	-0.160 (0.018)	-0.066 (0.022)**
Uric acid	18–49	315 *μmol/L*	5524	27 (1)	25 (1)
	50+	334 *μmol/L*	3506	30 (2)	23 (2)*
Systolic blood pressure	18–49	115.1 *mm Hg*	5594	3.8 (0.2)	3.9 (0.2)
	50+	134.1 *mm Hg*	3580	3.1 (0.3)	2.0 (0.4)
Diastolic blood pressure	18–49	72.9 *mm Hg*	5592	3.7 (0.2)	3.3 (0.2)
	50+	75.5 *mm Hg*	3580	2.0 (0.2)	2.0 (0.2)
Heart rate	18–49	73.4 *bpm*	5482	2.4 (0.3)	1.8 (0.2)
	50+	74.1 *bpm*	3493	2.2 (0.3)	1.2 (0.3)

* p < 0.01, ** p < 0.001, *** p < 0.0001, indicating significantly different beta coefficients for ln LAP and ln BMI.

† Lipoprotein data obtained only during phase 1 of NHANES III.

When the separate component measures that contribute to LAP (i.e., WC and TG concentration) were log-transformed and entered individually into predictive models, their standardized beta coefficients generally showed lesser or equivalent (p > 0.05) slopes compared to the slopes for LAP. The only exception was for the estimation of the ratio LDL cholesterol/Apo B among adults 18–49 years old. In this group the slope for ln TG alone [-0.160 (0.019)] was more steeply negative (p = 0.02) than the slope for ln LAP [-0.090 (0.017)].

## Discussion

The index described in this paper – the lipid accumulation product (LAP) – was developed in an effort to reflect the combined anatomic and physiologic changes associated with lipid overaccumulation in adults. Compared with BMI, LAP exhibited better correlations with lipid risk variables, uric acid concentration, and heart rate, but its correlation with blood pressure was roughly equivalent.

It is reasonable to speculate that the two LAP components – that is, enlarged abdominal fat depots and increased TG concentration – are each an indication that available lipid fuels have exceeded the individual's capacity to buffer and safely store this major form of acquired energy. Prior to 50 years old, the LAP appears to rise more slowly with age for women compared to men (Table 2). The women's relative delay of lipid overaccumulation is consistent with their greater amount of lower-body adipose tissue that confers increased buffering and storage capacity.

Whether an individual's excess lipid fuel appears eventually as an enlarged abdomen or as elevated circulating TG could be dictated in part by genes or by features of the individual's environmental circumstances. A special case, by way of an extreme example, might be the rare individual who is genetically disposed to extremely high TG concentrations (chylomicronemia). For the purpose of risk assessment in the general adult population, however, the alternative manifestations of lipid overaccumulation could be similarly informative. Regardless if the overaccumulation is marked by waist size, by TG concentration, or by both, the calculated value of LAP will be increased. In parallel with the LAP increments, excess lipid material will increasingly be deposited in nonadipose, "ectopic" tissues (e.g., liver, skeletal muscle, heart, blood vessels, kidneys, and pancreas) where it may adversely modify cellular metabolism, accelerate apoptosis (cell death), and interfere with cardiovascular control [10,11,31].

Ectopic lipid deposition is difficult to quantify directly, but an increased LAP value may indicate that various tissues or organs have become more vulnerable to injury from lipid overaccumulation. Lipoprotein particles with small diameters are more associated with disease risk than those with large diameters [32,33], and lipoprotein particle diameter is inversely associated with abdominal size [34]. Thus, LAP may effectively express disease risk through the hyperbolic relationship between LAP and its two component variables (Figure 2). The presence of an enlarged waist (implying a small lipoprotein particle size) allows the LAP value to increase rapidly with each unit increase in TG concentration. By contrast, the presence of a small waist (implying a large lipoprotein particle size) allows the LAP value to increase slowly with each unit increase in TG concentration. Although the NHANES III data set contains no direct measurement of particle sizes, our indirect estimate of LDL particle size (LDL cholesterol/ApoB) confirms that a small LDL particle size is better correlated with LAP than with BMI (Figure 3 and Table 4).

In contrast to an elevation in LAP value, an elevated BMI value (i.e., relative weight) is less specific in its anatomic or physiologic implications. Increased weight might represent enhancement of lean tissues, enlargement of the protective, subcutaneous adipose depots in the lower extremities [5,35-37], or systemic overload of fluid – changes that could be either salutary or simply secondary consequences of other disease processes. The commonly observed association of fluid overload with hypertension may explain the instances in which this study found blood pressure to be marginally better correlated with BMI than with LAP (Table 4 and Figure 3).

In order for LAP to gain a useful role in clinical medicine or epidemiology, at least three major questions remain to be addressed:

*1. Is LAP strongly predictive of major disease outcomes? *This preliminary analysis examined only intermediary outcomes (risk factors), and its data are entirely cross-sectional. However, a 20-year followup study of Swedish women reported that abdominal adiposity and elevated TG concentration were associated with increased risks of death from myocardial infarction and all causes, but that elevated BMI and cholesterol concentration were much weaker predictors [38]. In another prospective study from Scandinavia, post-menopausal women followed for 8.5 years demonstrated that the baseline combination of enlarged waist with elevated triglycerides – a dichotomous marker – was a very strong predictor of all-cause and cardiovascular mortality as well as the annual progression rate of aortic calcification [39]. More data collected prospectively in a variety of populations, including men, would help confirm that LAP – a continuous marker – has advantages over other simple indices for predicting the incidence of major diseases and mortality.

*2. Is it useful to monitor LAP values as an indicator of intervention effectiveness? *A recent report of intentional weight loss among overweight and obese Japanese women found that their exercise regimen and low-calorie diet were rewarded with a 37 percent reduction in TG concentration and a 27 percent reduction in truncal fat but only a 2 percent reduction in leg fat and a 12 percent reduction in BMI [40]. The authors also reported a direct correlation between changes in truncal fat and changes in fasting TG concentration or the number of heart disease risk factors, but that the changes in leg fat were *inversely *correlated with the changes in TG concentration or the number of heart disease risk factors. In another study, Italian men and women with diabetes were followed for two years after randomization to a physical activity counselling intervention [41]. Across six levels of aerobic energy expenditure, those who exercised more experienced significant reductions (p < 0.001) in waist circumference and circulating TG, but no reductions (p > 0.25) in either weight or BMI. Thus, the participants in both of these studies would have achieved a substantial reduction in LAP in association with improved cardiovascular risk factors, but their reduction in BMI was modest and less clearly associated with cardiovascular benefit. These observations from Asia and Europe demonstrate a potential advantage to using LAP as an intermediary variable by which to assess interventions against obesity-related risk.

*3. Is LAP a practical index for adoption by clinicians or epidemiologists? *Standardized waist measurements are highly reproducible [42], and they are arguably simpler and less expensive to obtain than well standardized weights and heights. The requirement of a venipuncture, however, could be a major obstacle for many potential participants or patients. The need for the fasting state could also represent a major inconvenience, although many persons accept the fasting condition when necessary for assessment of their glucose or lipid status. Of special importance in less developed economies, the laboratory cost of a single assay for TGs would be low compared with the costs of multiple assays for lipoproteins and an extensive biochemical panel.

## Conclusion

The cross-sectional associations with LAP demonstrated in this paper should be seen primarily as a demonstration of how the concept of lipid overaccumulation may be expressed in an adult population. The utility of LAP for research or as a practical tool for use in the community will depend on the degree to which LAP can be demonstrated to enhance prediction of disease incidence. Prospective data sets that include baseline information on WC and fasting TG concentration would be well suited to evaluate LAP as a predictor of cardiovascular outcomes and mortality.

## Abbreviations

ApoA1, apolipoprotein A1

ApoB, apolipoprotein B

BMI, body mass index

HDL, high-density lipoprotein

LAP, lipid accumulation product

LDL, low density lipoprotein

NHANES III, third National Health and Nutrition Examination Survey

## Competing interests

The author(s) declares that he has no competing interests.

## Authors' contributions

HK conceived of this study, performed the calculations, and drafted the manuscript.

## Pre-publication history

The pre-publication history for this paper can be accessed here:


