# Correction: The "lipid accumulation product" performs better than the body mass index for recognizing cardiovascular risk: a population-based comparison

**DOI:** 10.1186/1471-2261-6-5

**Published:** 2006-01-27

**Authors:** Henry S Kahn

**Affiliations:** 1 National Center for Chronic Disease Prevention and Health Promotion, CDC, Mail-stop K-10, 4770 Buford Highway, Atlanta, Georgia 30341-3717 USA

## Abstract

I have found an error in the Discussion section of my recent article [1]. While citing a prior work by Okura et al. [2], I incorrectly wrote that their weight-loss participants had "only a 2 percent reduction in leg fat". In context, the correct sentence should have said that their participants had "a 37 percent reduction in TG concentration, a 27 percent reduction in truncal fat, ***a 26 percent reduction in leg fat***, but only a 12 percent reduction in BMI."

I regret the inadvertent misquotation. My own data, my conclusions, and my admiration for Okura et al. are unchanged.
